# Estimated Energy Requirements of Infants and Young Children up to 24 Months of Age

**DOI:** 10.1093/cdn/nzab122

**Published:** 2021-09-29

**Authors:** Simona V Stan, Dominik Grathwohl, Lynda M O'Neill, Jose M Saavedra, Nancy F Butte, Sarah S Cohen

**Affiliations:** Société des Produits Nestlé S.A., Vevey, Switzerland; Clinical Research Unit, Nestlé Research, Société des Produits Nestlé S.A., Lausanne, Switzerland; Nestlé Institute of Health Sciences, Nestlé Research, Société des Produits Nestlé S.A., Lausanne, Switzerland; Société des Produits Nestlé S.A., Vevey, Switzerland; Department of Pediatrics, Johns Hopkins University School of Medicine, Baltimore, MD, USA; Department of Pediatrics, Baylor College of Medicine, Houston, TX, USA; EpidStrategies, a division of ToxStrategies, Inc., Cary, NC, USA

**Keywords:** energy requirements, doubly labeled water, infants, children, growth, total energy expenditure

## Abstract

**Background:**

Establishing energy requirements in infants and young children is important in developing age-appropriate diet recommendations but most published guidelines for energy requirements have 1 or more limitations related to the data underlying the calculations.

**Objective:**

To develop a comprehensive set of daily energy requirements for infants and young children aged 0–24 mo meeting the ideals of worldwide applicability to all healthy children based on the use of the doubly labeled water (DLW) technique to measure total energy expenditure (TEE), the use of recent, international growth charts, and calculation of values across a wide range of body weight.

**Methods:**

Daily estimated energy requirements (EERs) were calculated in 1-mo increments from 0 to 24 mo for boys, girls, and combined, using as inputs the following: *1*) TEE measured using the DLW technique, *2*) energy deposition estimates from the Institute of Medicine, and *3*) body weight values from the 25th to 75th percentiles from the 2006 WHO growth charts. EERs were combined for age groups 0 to <6, 6–8, 9–11, and 12–24 mo by averaging EERs from individual months. The EER calculations were supported by a systematic literature review and a meta-regression of existing studies.

**Results:**

Energy requirements naturally increase with age and are slightly higher in boys than in girls. The EERs derived in this study are similar to those in other recent international efforts.

**Conclusions:**

This updated set of EERs for infants and young children expand and improve upon the methodology used to establish previous published guidelines. These estimates have multiple potential uses including planning age-appropriate menus for the complementary feeding period, the development of foods that are more precisely targeted to the needs of infants and children at particular ages, and establishing macronutrient requirements within specific age groups based on a percentage of energy, such as dietary fat.

## Teaser

This article presents a newly updated and comprehensive set of estimated energy requirements for infants and young children with global applicability.

## Introduction

In growing infants and young children, daily energy requirements are estimated as the sum of *1*) total energy expenditure (TEE) at a level of physical activity consistent with normal development and *2*) energy for tissue deposition during growth at a rate consistent with health (1). In 1985, an expert committee of the FAO/WHO/UN University (UNU) established energy requirements in infants and children based on observations between 1940 and 1980 of actual food intakes in healthy infants and children with presumably normal growth (2). These estimates were arbitrarily increased by 5% to account for underestimation of intake, but the methodology involving reliance on observed intakes at a specific period of time has limitations inherent in the assumption that ad libitum intakes reflect optimal energy intakes and also in the assumption that feeding patterns are static over time rather than changing to reflect secular trends. Once the doubly labeled water (DLW) technique was developed and established as a more reliable method for total energy expenditure assessment, established energy requirements in infants and young children were challenged (3, 4, 5). Work by Butte et al. in 2005 also determined that the 1985 FAO/WHO/UNU energy requirements were overestimates and further identified differences in requirements for formula-fed versus breastfed infants (6). Although several currently available sets of energy requirements are being utilized worldwide, including those from the Institute of Medicine (IOM) in 2002, FAO/WHO/UNU in 2004, United Kingdom in 2011 (UK), and European Union in 2013 (EU) (1, 7, 8, 9), each lacks 1 or more elements of what might be considered ideal standards. Such ideal standards would include the use of the DLW technique to measure TEE, the use of universal international growth charts, presentation of energy needs across a wide spectrum of body weights rather than being limited to the 50th percentile, and applicability or generalizability to all healthy children worldwide. Therefore, this study was conducted in order to produce updated EERs for infants and toddlers that address the limitations of the existing guidelines and meet the ideal standards.

## Methods

The purpose of this study was to produce updated EERs for infants and toddlers aged ≤24 mo using prediction equations based on TEE established using the DLW method. These updated energy requirements also reflect universal, international growth standards (10) and cover a wider range of infant weights within normal limits than previous estimates (1, 7, 8, 9). As infants and toddlers grow rapidly, their energy requirements increase significantly over time; to accommodate this transition, this study provides energy requirements in 1-mo intervals. The methodology to derive the updated EERs is described below.

### DLW technique

The gold standard for the calculation of EERs is based on measures of TEE derived from studies using the DLW technique. In 2000, Butte et al. presented TEE data for healthy term infants in the USA aged ≤24 mo using the DLW technique (6). TEE estimates were presented for boys and girls separately over all feeding methods as well as within strata of breastfed and formula-fed infants for every 3 or 6 mo window through to the age of 24 mo.

We conducted a systematic literature review (SLR) in order to update a previous review presented in 2005 by Butte et al. (11) (**Supplemental Table 1**), which summarized published databases for TEE of infants by the DLW method (11). Our SLR identified articles published from 2000 to the present. A summary of the process is shown in **Supplemental Figure 1**. We utilized PubMed, EMBASE, Web of Science, Cochrane Trials, Cochrane Reviews, and clinicaltrials.gov to identify 119 unique abstracts for review of which 29 were selected for full-text review and 13 publications were found to be relevant. Of these 13 publications (12–23, 24), none presented more complete EERs over a range of ages among healthy infants with a variety of feeding types (formula-fed, breastfed, or mixed-fed) than the 2000 study by Butte et al. Four studies identified in our SLR estimated TEE for healthy infants (17, 18, 19, 22). Three of these studies had smaller sample sizes than the 76 infants included in the DLW study conducted by Butte et al. (N = 36, 44, and 10), and presented TEE values at limited ages (9 mo, 18 mo, and 10–40 mo [17, 18, 19]). The fourth study of healthy infants was larger (N = 162 total) and repeatedly measured TEE at ages 1.5, 3, 6, 9, and 12 mo (22). Three additional studies examined healthy infants limited to those who were exclusively breastfed (15, 20, 24). A further 3 studies estimated TEE at very young ages in premature or low birthweight infants (14, 16, 23), while another 2 studies examined TEE within categories of maternal body size (12, 21). The last identified study compared TEE estimates in infants at ages 3 and 12 mo among those with and without congenital heart disease (13). Of note, none of the newly identified articles provided estimates of TEE notably different from those presented in the comprehensive summary by Butte et al. in 2005.

### Prediction equation selection

A crucial input to any equation that predicts energy requirements is a measure of TEE. The DLW technique has been established as a reliable method for estimating TEE in infants (25–27, 28). Two different sets of prediction equations for TEE based on studies utilizing the DLW technique have been used in establishing energy requirements in a variety of guidelines developed worldwide. The first model, applied by the FAO/WHO/UNU and based on work by Butte, fits different equations for children aged <12 mo and those aged 1 y and older (8). The FAO/WHO/UNU model also provides separate equations for different feeding types for children aged <12 mo to predict TEE. The FAO/WHO/UNU model has been used to develop EERs for several international guidelines including in the UK (7), EU (9), and China (29) and is calculated as follows for infants aged ≤12 mo:

(1) $$\begin{equation*}
{\rm{All infants\!:}}\,\,{\rm{TEE}}\,\,[ {{\rm{kcal/d}}} ] = 88.3*{\rm{weight}}\,\,[ {{\rm{kg}}} ] - {\rm{95}}{\rm{.4}}
\end{equation*}$$

(2) $$\begin{equation*}
{\rm{Breastfed\!:}}\,\,{\rm{TEE}}\,\,[ {{\rm{kcal/d}}} ]= 92.8*{\rm{weight}}\,\,[ {{\rm{kg}}} ] - {\rm{152}}
\end{equation*}$$

(3) $$\begin{equation*}
{\rm{Formula - fed\!:}}\,{\rm{TEE}}\,\,[ {{\rm{kcal/d}}} ] = 82.6*{\rm{weight}}\,\,[ {{\rm{kg}}} ] - {\rm{29}}
\end{equation*}$$

A second equation to predict TEE, developed by the IOM and also based on work by Butte, applies to all infants aged 0–36 mo regardless of feeding method (1) as follows:

(4) $$\begin{equation*}
{\rm{TEE }}\,\,[ {{\rm{kcal/d}}} ] = 89*{\rm{weight }}\,\,[ {{\rm{kg}}} ] - {\rm{100}}
\end{equation*}$$

The output of the FAO/WHO/UNU and IOM equations is very similar for infants. Thus, for the updated EERs calculated and presented in this study, the single IOM equation was selected due to its coverage of a wider age range. Systematic differences between boys and girls are consequences of the sex differences in weight.

### Growth chart selection

Calculating EER requires input information on the weight of infants and children at a variety of ages. Some of the existing sets of energy requirement guidelines have utilized outdated growth charts or charts limited to one country. For example, the IOM DRIs (1) were calculated for the USA and Canada using CDC growth charts based on 5 national health examination surveys conducted between 1963 and 1994 in the USA (30). For the current study, the WHO 2006 growth charts were used both for their more recently updated data and due to their international coverage, as these charts represent growth data from healthy infants and children from Brazil, Ghana, India, Norway, Oman, and the USA (10). The WHO 2006 growth charts have also been utilized in the estimates of energy requirements created in the UK (7) and EU (9). Additionally, most presentations of EERs have been limited to infants at the 50th percentile of growth, but we sought to provide estimates over a wider range of infant weights. Thus, we utilized multiple weight values between the 25th and 75th percentiles in our calculation of EERs.

### Calculation of EERs

Because infants and young children are growing rapidly, especially in the first 6 mo of life, energy deposition must be added to the TEE to fully capture energy requirements. Thus, the calculation of EERs for infants and children aged 0–24 mo was performed in this study using the following equation where (89*weight [kg] – 100) represents the TEE plus a growth allowance. The precision of the TEE formula is ±109 kcal/d (SE of the estimation) (11).

(5) $$\begin{equation*}
{\rm{EER }}\,\,[ {{\rm{kcal/d}}} ] = ( {89*{\rm{weight }}\,\,[ {{\rm{kg}}} ] - {\rm{100}}} ) + {\rm{energy \, deposition}}
\end{equation*}$$

The input energy deposition values (kcal/d) were obtained from the IOM report (1), which computed energy deposition from rates of protein and fat deposition observed in a longitudinal study of infants aged ≤24 mo (6). Energy deposition values were available for ages 3, 6, 9, 12, 18, and 24 mo. Linear interpolation was used to determine values for each month of age. The input weight values were obtained from the WHO 2006 growth standards, as described above, separately for boys and girls, for the 25th, 50th, and 75th percentiles at each month of age. After EERs were calculated separately for boys and girls at the 25th, 50th, and 75th percentiles for weight, combined EERs over sex were calculated by averaging the EERs for boys and for girls at each percentile and month of age.

### Age windows

The EERs presented here were calculated at each month of age from 0 to 24 mo and then subsequently grouped according to standard categories. The upper limit of age of 24 mo was selected to correspond with the upper age limit of the complementary feeding period defined by the WHO (31, 32).

### Meta-regression of data obtained from 2000–present SLR

The values for TEE obtained via the DLW method as well as mean weight as identified in the literature from the updated SLR (covering the period 2000 to present) were utilized in a random-effects meta-regression to compute a summary estimate of TEE. The meta-regression is a linear regression of the study effect sizes (TEE) on study-level covariates (infant weight) and investigates whether between-study heterogeneity can be explained by the study-level covariates. Assumptions for the meta-regression are the same as for other random-effects models, namely, the distribution of the random effects and the error terms follow a normal distribution with mean zero and constant variance. The error terms are also assumed to be independent of each other, and independent of the random effects. In order to be comparable to previous models that have focused on healthy infants, studies of preterm or ill infants were not included in the meta-regression. The meta-regression model utilized data points obtained from each study at all available ages; for example, a study that reported TEE and weight values at ages 3, 6, 9, and 12 mo was included in the model as 4 data points, 1 at each age. The meta-regression model included only 1 independent variable (weight). The intercept and β-coefficient for weight from the meta-regression model were used to compute estimates of TEE at weight values from the WHO 2006 at the 25th, 50th, and 75th percentiles for boys and girls separately over the age range of 0–24 mo. These predicted TEE values were compared with the values predicted by the IOM model graphically.

### Ethics

The meta-regression uses summary data from the public domain and does not use subject-level data and hence is fully aligned with the general data protection regulation (GDPR) of the EU (2016): https://eur-lex.europa.eu/eli/reg/2016/679/oj.

## Results

A summary of EERs is shown for categories of age from 0 to 24 mo in infants and toddlers in **Table 1**, separately for boys and girls, as well as both sexes combined at the 25th, 50th, and 75th weight percentiles. Energy requirements naturally increase with age and are slightly higher in boys than in girls. The complete set of EERs at each month of age from 0 to 24 mo for the selected weight percentiles are shown in **Table 2** and **Supplemental Figure2** (boys), **Table 3** and **Supplemental Figure 3** (girls), and **Table 4** and **Supplemental Figure 4** (combined boys and girls). EERs over a wider range of body weight percentiles (5th to 95th percentiles) for boys, girls, and combined are shown in **Supplemental Tables 2–4** and show similar patterns with increasing values with rising age and slightly higher values in boys.

**TABLE 1:** Estimated energy requirements (EERs) for infants and toddlers aged 0–24 mo at 25th, 50th, and 75th WHO 2006 percentiles for body weight^1^

	EER (kcal/d) at 50th percentile (25th percentile, 75th percentile) for body weight		
Age groups	Boys	Girls	Combined
0–6 mo	559 (517, 604)	512 (471, 557)	536 (494, 580)
6–8 mo	668 (615, 724)	615 (561, 674)	641 (588, 699)
9–11 mo	742 (684, 805)	680 (621, 746)	711 (653, 775)
12–24 mo	899 (828, 975)	836 (764, 917)	868 (796, 946)

1 Source of weights at each percentile is WHO (10).

**TABLE 2:** Estimated energy requirements (kcal/d) for boys, aged 0–24 mo at 25th, 50th, and 75th WHO 2006 percentiles for body weight

	Weight percentile^1^		
Month	25th	50th	75th
0	352	381	411
1	445	480	517
2	553	594	637
3	565	609	655
4	533	579	628
5	570	618	670
6	601	652	706
7	609	662	719
8	634	689	747
9	658	714	775
10	692	750	812
11	709	769	833
12	729	790	856
13	741	804	871
14	759	824	893
15	777	843	914
16	794	862	935
17	812	881	956
18	829	900	976
19	846	918	996
20	862	936	1016
21	879	955	1037
22	896	973	1057
23	912	991	1077
24	928	1009	1097

1 Source of weights at each percentile is WHO (10).

**TABLE 3:** Estimated energy requirements (kcal/d) for girls, aged 0–24 mo at 25th, 50th, and 75th WHO 2006 percentiles for body weight

	Weight percentile^1^		
Month	25th	50th	75th
0	322	349	377
1	405	439	474
2	484	523	565
3	513	555	602
4	494	540	590
5	526	574	628
6	554	605	661
7	553	607	666
8	577	633	695
9	600	658	721
10	624	684	749
11	642	703	771
12	661	724	794
13	676	741	812
14	694	760	834
15	712	780	855
16	730	799	876
17	747	818	896
18	765	837	917
19	782	856	938
20	799	874	958
21	816	893	979
22	833	912	999
23	851	931	1020
24	868	950	1041

1 Source of weights at each percentile is WHO (10).

**TABLE 4:** Estimated energy requirements (kcal/d) for boys and girls combined, aged 0–24 mo at WHO 2006 25th, 50th, and 75th percentiles for body weight

	Weight percentile^1^		
Month	25th	50th	75th
0	337	365	394
1	425	459	496
2	519	558	601
3	539	582	629
4	513	559	609
5	548	596	649
6	577	629	684
7	581	635	692
8	606	661	721
9	629	686	748
10	658	717	781
11	675	736	802
12	695	757	825
13	708	772	842
14	726	792	863
15	744	811	884
16	762	830	905
17	779	849	926
18	797	868	946
19	814	887	967
20	831	905	987
21	848	924	1008
22	865	942	1028
23	881	961	1048
24	898	980	1069

1 Source of weights at each percentile is WHO (10).

**FIGURE 1:**
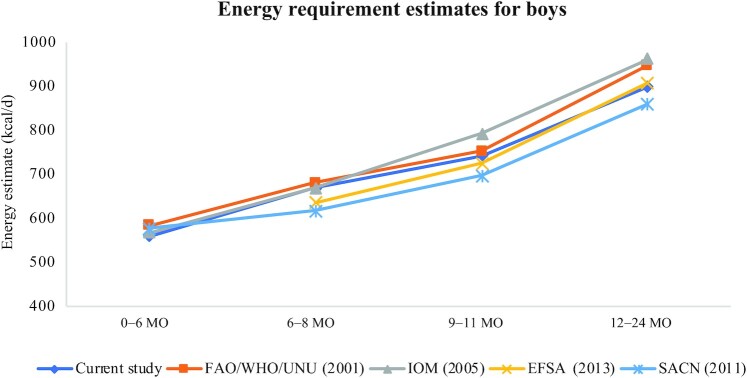
Comparison of estimated energy requirements across published international guidelines for boys. EFSA, European Food Safety Authority; IOM, Institute of Medicine; SACN, Scientific Advisory Committee on Nutrition; UNU, UN University.

**FIGURE 2:**
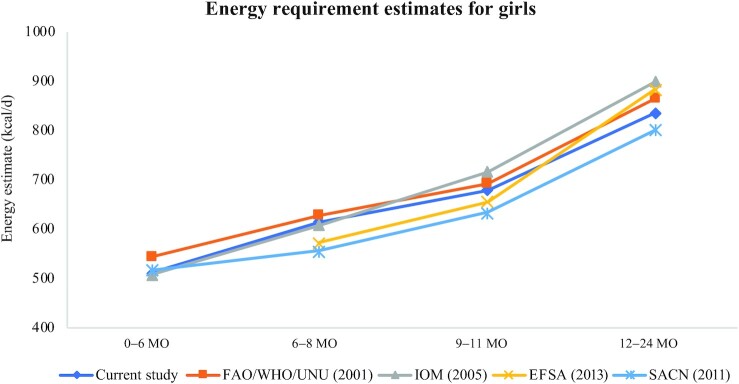
Comparison of estimated energy requirements across published international guidelines for girls. EFSA, European Food Safety Authority; IOM, Institute of Medicine; SACN, Scientific Advisory Committee on Nutrition; UNU, UN University.

**FIGURE 3:**
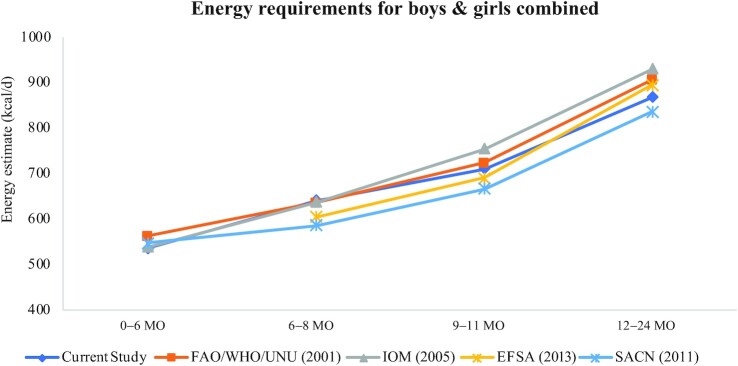
Comparison of estimated energy requirements across published international guidelines for boys and girls combined. EFSA, European Food Safety Authority; IOM, Institute of Medicine; SACN, Scientific Advisory Committee on Nutrition; UNU, UN University.

The EERs derived in this study closely follow those in other recent international efforts (**Figures 1****–****3**) including reports from the UK, EU, IOM, and FAO/WHO/UNU (1, 7–9) despite variations in the underlying prediction equations used for TEE, source of DLW data, and the growth standards used to obtain infant weights. EERs combined over boys and girls at the 50th WHO 2006 percentile of weight are shown for different international sources in **Table 5**. The estimates from the current study tend to fall in the middle at each age point with the European Food Safety Authority (EFSA) and the UK Scientific Advisory Committee on Nutrition (SACN) values being slightly lower, particularly before 12 mo, and the IOM and FAO/WHO/UNU estimates being slightly higher. Russian energy requirements were calculated from the energy norms provided in the “Standards of physiological needs for energy and nutrients for various groups of the population of the Russian Federation” (33) applied to the weights at the 50th percentile from the WHO 2006 growth charts; the EERs calculated for the Russian population are notably higher than the other guidelines. Excluding the Russian guidelines which produced higher EERs than the other international guidelines, differences in EERs were generally similar across guidelines; for example, for boys, the maximum difference across all guidelines was 62, 95, and 101 kcal/d, at 6–8, 9–11, and 12–24 mo, respectively, whereas for girls, the maximum differences were 72, 82, and 98 kcal/d, respectively.

**TABLE 5:** Summary of estimated energy requirements (EERs) across international guidelines for boys and girls combined at the 50th percentile of weight

	Calculation of EER		EER in kcal/d^2^			
Guidelines	Predictive equation for TEE^1^	Source of body weight data	0 to <6 mo	6–8 mo	9–11 mo	12–24 mo
Current study	IOM	WHO 2006	536	641	711	868
FAO/WHO/UNU	FAO/WHO/UNU	WHO 1994	563	635	723	907
IOM	IOM	CDC 2000	538	638	755	930
EFSA	FAO/WHO/UNU	WHO 2006	Unavailable	604	690	896
SACN	FAO/WHO/UNU	WHO 2006	548	587	666	837
China	FAO/WHO/UNU	Chinese growth and development reference standards 2009	582	662	729	851
Russia	Unavailable	WHO 2006	667	869	968	1200

1 WHO models: breastfed: TEE [kcal/d] = 92.8*weight [kg] – 152; formula-fed: TEE [kcal/d] = 82.6*weight [kg] – 29. IOM model: TEE [kcal/d] = 89*weight [kg] – 100. EFSA, European Food Safety Authority; IOM, Institute of Medicine; SACN, Scientific Advisory Committee on Nutrition; TEE, total energy expenditure; UNU, UN University.

2 From the published guidelines from FAO/WHO/UNU (8), IOM (1), EFSA (9), and SACN (7), averages of estimated energy requirements by age group and sex at the 50th percentile for weight were abstracted and then averaged over sex to produce the combined summary estimates for each age category shown. Data on energy requirements in China were based on the Chinese DRIs (2013) and for infants aged <12 mo, the 2009 growth charts (median values for body weights for the various age groups, for boys and girls combined) were applied. For infants 0–11 mo, Russian energy requirements were calculated from the energy norms provided in the guidance document and then applied to the sex-averaged 50th percentile body weight values from the WHO 2006 growth charts (10). For children aged 12–24 mo, the energy requirement of 1200 kcal/d was taken directly from the Russian guidance document (13).

Comparisons of predicted TEE from the IOM model and the meta-regression using the updated SLR data are shown in **Supplemental Figures 5**and**6**. The model parameters (β and intercept) are similar between the IOM and meta-regression models, and the predicted values are nearly identical for both boys and girls in the youngest infants. The predicted values begin to diverge slightly after ∼9 mo of age but remain very close between the 2 models through to 24 mo. Of note, the meta-regression is based on aggregate data whereas the IOM model was based on individual data so these comparisons should be interpreted as general validation of the IOM model estimates rather than a strict comparison.

## Discussion

This study presents EERs for infants and young children aged ≤24 mo utilizing, for the first time collectively, a robust and updated set of inputs including the reliance on the DLW technique to measure TEE, the use of recent, international growth charts, and presentation of energy needs across a wider spectrum of body weights. The specific inputs into the calculation of these updated EERs are first, mathematical prediction equations used in the IOM recommendations; second, body weights for a range of weight percentiles from the more recent and internationally recognized 2006 WHO growth charts; and third, estimates of energy deposition determined by the IOM based on rates of protein and fat deposition.

Two published and validated sets of prediction equations for the calculation of TEE utilizing underlying data derived from studies using the DLW technique are available, including a model from the IOM and a model from the FAO/WHO/UNU. The 2 models provide similar results but with slightly different parameterizations. The first model, from the IOM, utilized 320 measurements derived from studies of DLW in infants and young children to fit a single equation to predict TEE (1). Weight was found to be the best predictor of TEE (factors such as infant sex or infant height did not make a statistically independent contribution), and thus, the inputs for the IOM model are simply weight values alone. The IOM model further does not differentiate between feeding types as the authors showed that differences in TEE between breastfed and formula-fed infants decreased after the age of 1 y; further, even for infants <1 y, the difference in TEE between formula-fed and breastfed infants was a maximum of only 12% at the age of 3 mo with the difference diminishing rapidly thereafter (11). The second model, from the FAO/WHO/UNU, includes prediction equations for all infants as well as separate regression equations for TEE based on feeding type (breastfed versus formula-fed). Similar to the IOM model, the FAO/WHO/UNU equations also use infant weight values as inputs. Using an updated SLR of published reports of TEE estimated using the DLW method, we used meta-regression to produce an additional set of estimates for TEE as validation of the IOM estimates. These estimates were confirmatory for the general trajectory and range of values from the IOM model.

Current guidelines for energy estimates utilized internationally all rely on studies using the DLW technique with most using the same reference set of TEE data as reported by Butte et al. (6). The energy requirement estimates vary by country with some, Brazil for example, adopting the 2002 IOM guidelines, and others establishing their own sets of estimates. China utilized the FAO/WHO/UNU prediction equations with separate estimates for mixed-fed versus breastfed infants, the database of DLW created by Butte et al. (6), and Chinese growth and development reference standards to produce estimates for infants ≤12 mo at the median weight (29, 34). The UK and EU estimates were both produced using the 2006 WHO growth standards as well as the 2004 FAO/WHO/UNU prediction equations for TEE; the UK estimates used separate equations for each feeding type (7) whereas the EU report concluded that the equation for breastfed infants also applies to formula-fed infants due to recent changes in the composition of infant formula to include a protein to energy ratio closer to human milk (9). The 2004 FAO/WHO/UNU guidelines utilized prediction equations for formula-fed and breastfed infants separately as presented in the 2000 report by Butte et al. utilizing the DLW method (6) as well as body weight measures from the 1994 WHO publication, with the measure of body weight coming from studies conducted as far back as the 1980s (8). Unlike the other guidelines presented above, the IOM 2002 report produced EERs for each month of age from 0 to 36 mo, utilized a single prediction equation regardless of feeding type, and included body weight values taken from CDC reference charts for the USA (1).

Uniformly, the existing sets of estimates were provided only at the 50th weight percentile for a given age, a limitation that this study sought to overcome by producing estimates over a wider range of weight percentiles at each age. As demonstrated in this work, despite differences in the underlying inputs for the calculation of EERs in terms of underlying mathematical models of TEE and source of body weight data, the resulting sets of energy estimates at the 50th percentile of body weight are quite similar to one another with the exception of the higher estimates from Russia. Excluding Russian guidelines from the comparison, our combined EERs at the 50th percentile for weight for 0 to <6 mo is 536 kcal/d with other guidelines ranging from 538 (IOM) to 563 (FAO/WHO/UNU) kcal/d. The EERs from our study and other international guidelines for 6–8 mo, 9–11 mo, and 12–24 mo all fall similarly within a small range (587–638, 666–755, and 837–930 kcal/d, respectively).

The estimates presented here expand upon other published guidelines by providing estimates over a larger range of body weights (25th to 75th percentile) and are also computed both at each month of age from 0 to 24 mo and also within age categories that are applicable to other work. For example, the EERs in the specific age windows (6–8, 9–11, and 12–24 mo) can be used to plan age-appropriate menus for the complementary feeding period. An understanding of the precise energy requirements at a specific age will also allow for the development of foods that are more precisely targeted to the needs of infants and young children at particular ages. Finally, these updated EERs will enable the establishment of macronutrient requirements within specific age groups based on a percentage of energy, such as for dietary fat.

As our primary results (Table 1), we have chosen to show EERs at the 25th, 50th, and 75th percentiles of body weight, and these will be the basis for future work designing age-appropriate menus for the complementary feeding period. These percentiles were selected to represent the normal range of body weights as the 75th percentile for body weight is recognized as a cut-off for overweight (35) and the 25th percentile is equally spaced below the median. “Failure to thrive” is defined as low when below the 5th percentile (36, 37), but in developing diets for infants in this time period, the 25th percentile is a more conservative cut-point to use in focusing on children with adequate growth. Although we have presented EERs for a wider range of percentiles of body weight in Supplemental Tables 2–4, caution should be used in the interpretation and utilization of the values calculated at the most extreme percentiles (namely the 5th and 95th) as those are based on very sparse data. Level of physical activity was not specified in the calculation of TEE based on an assumption of the limited range of activity observed at any given age ≤24 mo.

Our primary presentation is EER estimates for breastfed and formula-fed infants combined as we utilized the IOM model which does not incorporate feeding method. Although we believe this is an appropriate choice of model given the high prevalence of mixed feedings globally (38, 39) and the similarities in the outputs of the FAO/WHO/UNU and IOM models, we acknowledge that there are differences in the proportion of infants who are breastfed, mixed-fed, and formula-fed across and within populations, and thus calculations for breasted and formula-fed infants could vary. We have therefore additionally presented EER estimates separately for breastfed and formula-fed infants at the 25th, 50th, and 75th percentiles of weight in **Supplemental Tables 5, 6**,and**7** for the first 12 mo of life. These estimates are similar to those presented in our primary analysis with the breastfed estimates slightly lower than the combined estimates and the formula-fed estimates slightly higher. Feeding type differences become smaller as age increases with minimal differences by the age of 12 mo.

The EERs presented in this study show several improvements over existing references for energy requirements in young children. First, the estimates use the internationally representative 2006 WHO growth curves. The WHO growth standards were derived from healthy breastfed infants living in optimal environmental conditions (40). Second, the energy recommendations have been expanded beyond the 50th percentile of weight to provide EERs across a range of infant weights (25th to 75th percentiles). These improvements expand the potential uses of the EERs and also ensure they are based on standard body weights. There are also limitations of the approach used to calculate these estimates. Although validated in infants and young children, there is still uncertainty around TEE estimates using the DLW technique as the number of available DLW studies in infants remains small and data are limited especially from developing countries. A second limitation is related to the assumptions for energy deposition values in our derivation of EERs. Energy deposition values were available from the age of 0.5–24 mo from IOM and then we determined monthly values by linear interpolation. The FAO/WHO/UNU report notes that energy demands for growth constitute about 35% of the total energy requirement during the first 3 mo of life, this proportion is halved in the next 3 mo (i.e., to about 17.5%), and further reduced to one-third of that during the ensuing 6 mo (i.e., to <6%) and to only 3% at 12 mo; energy for growth falls to <2% of daily requirements in the second year (8). Thus, although the interpolated values used in our EER calculations are based on aggregate data with variation by age and we further compute EERs across a range of weight percentiles to better address variability in energy demands for growth in the first year, there is no further consideration in our calculations for variability that may exist in energy demands for growth between children at a given age and weight percentile.

In conclusion, the updated EERs for infants and toddlers up to the age of 24 mo presented in this study utilize, for the first time collectively, a robust and updated set of inputs including the reliance on the DLW technique to measure TEE, the use of international growth charts, and presentation of energy needs across a wide spectrum of body weights. In today's economy, the food industry manufactures products that are sold internationally. Thus, these guidelines, which rely on international standards, can be used to develop age-appropriate feeding standards and to guide product formulation and menu planning that are globally relevant.

## Supplementary Material

nzab122_Supplemental_File

## Data Availability

The data used in this manuscript, the code book, and the analytic code can be made available upon request pending application and approval.

## Notes

This work was funded by Nestlé, Vevey, Switzerland.

Author disclosures: SVS, LMO'N, and DG are employees of Nestlé (funding source). JMS was an employee of Nestlé when the work was initiated. NFB was a compensated consultant for Nestlé until June 2020. SSC is a currently compensated consultant for Nestlé.

Supplemental Tables 1–7 and Supplemental Figures 1–6 are available from the “Supplementary data” link in the online posting of the article and from the same link in the online table of contents at https://academic.oup.com/cdn/.

Present address for JMS: Department of Pediatrics, Johns Hopkins University School of Medicine, Baltimore, MD, USA.

Abbreviations used: DLW, doubly labeled water; EER, estimated energy requirement; EU, European Union; IOM, Institute of Medicine; SLR, systematic literature review; TEE, total energy expenditure; UNU, UN University.
