# Supplementary material for: Estimated Energy Requirements of Infants and Young Children up to 24 Months of Age
Source: Curr Dev Nutr. 2021 Sep 29;5(11):nzab122. doi: 10.1093/cdn/nzab122 (PMC8575726; doi:10.1093/cdn/nzab122)
Supplement: nzab122_Supplemental_File [file nzab122_supplemental_file.pdf]

Online Supporting Material

Supplemental Table 1. Descriptive information [generally mean (standard deviation)] for total energy expenditure (TEE) of infants by doubly labelled water method published since 2000[1]

Reference (Country)	Population Description	N	Population	Age (months)	Weight (kg)	Height (cm)	FFM (kg)	TEE (kcal/day)	TEE (kJ/day)	TEE (kcal/kg/day)	Included in Meta-Regression
First Year of Life											
Salazar. 2000 (Chile)[20]	Exclusively breastfed infants	17	Low SES	1 (0.13)	4.51 (0.40)	54.3 (1.6)	NA	288 (75)	1205 (312)	64	Y
Olhager, 2003 (Sweden)[23]	Pre-term and full-term infants	8	Preterm	2 (0.33)	3.35 (0.25)	52 (1.0)	NA	253 (26)	1057 (110)	76	N
		9	Full-term	0.26 (0.1)	3.89 (0.56)	54 (2.0)	NA	237 (60)	993 (251)	61	Y
Reichman, 2003 (United Kingdom; Australia)[22]	Healthy full-term infants	72	NA	1.32 (0.26)	4.75 (0.63)	55.7 (2.4)	3.92 (0.56)	335 (103)	1402 (431)	71	Y
		111	NA	2.79 (0.36)	5.98 (0.68)	60.2 (2.2)	4.55 (0.53)	445 (112)	1862 (469)	74	Y
		64	NA	6.18 (0.36)	7.92 (0.86)	67.5 (2.6)	5.87 (0.80)	631 (124)	2640 (519)	80	Y
		41	NA	9.04 (0.23)	8.89 (1.11)	71.6 (2.8)	6.61 (0.94)	734 (158)	3071 (661)	83	Y
		34	NA	12.23 (0.46)	10.09 (1.18)	76.4 (3.4)	7.62 (0.90)	872 (191)	3648 (799)	86	Y
Stunkard, 2004 (United States)[21]	Infants of obese and lean mothers	18	Obese mothers	3	6.0 (0.8)	61.1 (2.2)	NA	414 (75)	1732 (312)	69	Y
		18	Lean mothers	3	6.1 (0.6)	61.4 (1.9)	NA	419 (92)	1753 (384)	69	Y
Haisma, 2006 (Brazil)[24]	Exclusively breast-fed infants	32	Middle SES	8.7	8.7 (1.0)	70.7 (2.5)	6.2 (0.6)	542	2267	62	Y
		33	Low SES	8.7	8.2 (1.1)	69.3 (2.9)	5.9 (0.9)	628	2627	77	Y
Torine, 2007 (United States)[14]	Extremely preterm infants during and after an episode of sepsis	10	Septic period: Sepsis	0.47 (0.20)	0.91 (0.25)	NA	NA	87 (23)	364 (96)	96 (25)	N
		10	Septic period: Control	0.67 (0.23)	1.03 (0.33)	NA	NA	69 (12)	289 (50)	67 (12)	N
		10	Recovery period: Sepsis	1.17 (0.37)	1.32 (0.44)	NA	NA	73 (22)	305 (92)	55 (17)	N
		10	Recovery period: Control	1.2 (0.23)	1.34 (0.41)	NA	NA	92 (20)	385 (84)	69 (15)	N
Guilfoy, 2008 (United States)[16]	Extremely low birthweight infants and	10	Extremely low birthweight	2.27 (0.30)	2.0 (0.20)	NR	0.002 (0.0002)	178 (44)	744 (184)	89	N
		14	full-term	0.08 (0.03)	3.4 (0.50)	NR	0.0027 (0.0005)	197 (65)	824 (272)	58	Y

Reference (Country)	Population Description	N	Population	Age (months)	Weight (kg)	Height (cm)	FFM (kg)	TEE (kcal/day)	TEE (kJ/day)	TEE (kcal/kg/day)	Included in Meta-Regression
	full term infants										
Gondolf, 2012 (Denmark)[17]	Infants on a western diet	35	NA	9.03 (0.2)	9.4 (1.0)	73.2 (3.2)	NA	689 (28)	2881 (118)	309 (46)	Y
Nielsen, 2013 (Scotland)[15]	Exclusively breast-fed infants	17	Male	3.50 (0.25)	6.81 (0.77)	62.40 (2.10)	4.92 (0.55)	484 (81)	2024 (340)	71	N
		19	Female	3.61 (0.37)	6.41 (0.69)	61.30 (2.20)	4.64 (0.46)	441 (41)	1846 (173)	69	N
		36	Total	3.54 (0.32)	6.60 (0.74)	61.80 (2.20)	4.77 (0.52)	461 (66)	1930 (276)	70	Y
		16	Male	5.62 (0.35)	7.88 (0.81)	66.30 (2.00)	5.60 (0.62)	583 (115)	2440 (480)	74	N
		17	Female	5.71 (0.23)	7.46 (0.83)	65.50 (2.30)	5.22 (0.58)	509 (59)	2130 (249)	68	N
		33	Total	6.12 (0.30)	7.66 (0.83)	65.90 (2.20)	5.40 (0.62)	545 (97)	2280 (405)	71	Y
Trabulsi, 2015 (United States)[13]	Infants with and without congenital heart disease	12	Healthy Infants	3.09 (0.28)	6.15 (0.69)	NA	4.50 (0.50)	NA	NA	NA	Y
		15	CHD infants	3.14 (0.41)	5.87 (0.94)	NA	4.54 (0.65)	439 (138)	1837 (577)	75	N
		12	Healthy infants	NA	9.71 (0.81)	NA	6.78 (0.33)	706 (91)	2954 (381)	73	Y
		11	CHD infants	12.43 (0.83)	9.15 (1.18)	NA	6.92 (0.79)	767 (124)	3209 (519)	84	N
Zinkel, 2016 (United States)[12]	Children with lean mothers and children with overweight or obese mothers	2	BMI1	3.00	5.90 (1.00)	62.00 (3.00)	NR	396 (25)	1657 (105)	67	Y
		31	BMI2	3.00	6.00 (0.70)	61.00 (2.00)	NR	405 (76)	1695 (318)	68	Y
		3	BMI3	3.00	6.30 (0.70)	62.00 (2.00)	NR	449 (106)	1879 (444)	71	Y
		6	BMI4	3.00	6.10 (0.70)	61.00 (3.00)	NR	434 (83)	1816 (347)	71	Y
Second Year of Life											
Eriksson, 2012 (Sweden)[18]	Healthy infants	21	Female	18.7 (0.40)	11.7 (1.40)	83 (3.0)	8.50 (0.86)	939 (100)	3929 (418)	80	N
		23	Male	18.5 (0.47)	12.1 (1.10)	83 (2.0)	8.66 (0.72)	970 (98)	4058 (410)	80	N
		44	Total	18.6 (0.47)	11.9 (1.20)	83 (3.0)	8.58 (0.78)	956 (96)	4000 (402)	80	Y
Ciampolini, 2013 (Italy)[19]	Healthy infants	10	At recruitment	21 (7.3)	11.1 (2.4)	83.4 (8.1)	NA	889 (77)	3720 (322)	80.1 (6.9)	Y
		10	After 50 days	22.7 (7.5)	11.4 (2.3)	85.3 (8.0)	NA	773 (114)	3234 (477)	67.8 (10.0)	Y
Stunkard, 2004 (United States)[21]	Infants of obese and lean mothers	24	Obese Mothers	24	12.3 (1.4)	89.3 (2.9)	NA	1004 (134)	4202 (561)	82	Y
		21	Lean Mothers	24	12.1 (0.9)	89.9 (2.9)	NA	1054 (102)	4410 (427)	87	Y

[1] Butte et al (2000)[6] published in *Am J Clin Nutr* was not included in this table as it is already summarized in Table 1 of Butte et al. 2005[11] in *Public Health Nutrition*
TEE- total energy expenditure; FFM - fat free mass; FM - fat mass; SEP - sepsis group; CON - control group; F - female; M - male; HI - healthy infants; CHD - congenital heart disease; BMI1 - BMI percentile 0-4.9; BMI2- BMI percentile 5-84.9; BMI3 - BMI percentile 85-94.9; BMI4 - BMI percentile 95-100

Online Supporting Material

Supplemental Table 2. Estimated energy requirements (kcal/d) for boys, ages 0-24 months at 5th to 95th WHO 2006 percentiles for body weight

	Weight percentile[1]								
**Month**	**5th**	**10th**	**15th**	**25th**	**50th**	**75th**	**85th**	**90th**	**95th**
0	315	328	338	352	381	411	428	440	458
1	399	416	427	445	480	517	539	553	576
2	500	519	533	553	594	637	661	678	703
3	507	528	543	565	609	655	682	700	728
4	471	493	509	533	579	628	656	675	705
5	505	529	545	570	618	670	699	720	751
6	534	558	575	601	652	706	737	758	790
7	539	565	583	609	662	719	750	773	806
8	561	588	606	634	689	747	780	803	838
9	583	610	629	658	714	775	809	833	869
10	614	642	662	692	750	812	847	872	909
11	630	659	679	709	769	833	869	895	933
12	647	677	697	729	790	856	893	919	959
13	657	687	709	741	804	871	910	936	977
14	674	704	726	759	824	893	932	960	1002
15	689	721	743	777	843	914	954	982	1026
16	705	737	760	794	862	935	976	1005	1049
17	720	753	776	812	881	956	998	1027	1073
18	735	769	793	829	900	976	1019	1050	1096
19	750	785	809	846	918	996	1041	1072	1119
20	765	800	825	862	936	1016	1062	1094	1143
21	780	816	841	879	955	1037	1083	1116	1166
22	794	831	856	896	973	1057	1104	1138	1189
23	808	846	872	912	991	1077	1126	1160	1212
24	823	861	888	928	1009	1097	1147	1182	1236

[1] Source of weights at each percentile: WHO Child Growth Standards: Length/height-for-age, weight-for-age, weight-for-length, weight-for-height and body mass index-for-age: Methods and development. Geneva, 2006.

Online Supporting Material

Supplemental Table 3. Estimated energy requirements (kcal/d) for girls, ages 0-24 months at 5th to 95th WHO 2006 percentiles for body weight

	Weight percentile[1]								
**Month**	**5th**	**10th**	**15th**	**25th**	**50th**	**75th**	**85th**	**90th**	**95th**
0	286	299	308	322	349	377	393	404	421
1	362	377	388	405	439	474	495	509	531
2	434	452	465	484	523	565	589	605	631
3	457	477	491	513	555	602	628	647	675
4	434	456	471	494	540	590	619	639	670
5	462	485	501	526	574	628	658	680	713
6	487	511	528	554	605	661	694	716	751
7	484	509	526	553	607	666	700	723	760
8	506	531	550	577	633	695	730	755	793
9	526	553	571	600	658	721	758	784	824
10	548	575	594	624	684	749	787	814	856
11	563	591	611	642	703	771	810	838	881
12	580	609	629	661	724	794	834	863	907
13	594	623	644	676	741	812	854	883	929
14	610	640	661	694	760	834	876	907	954
15	626	656	678	712	780	855	899	930	978
16	641	673	695	730	799	876	920	952	1002
17	657	689	712	747	818	896	942	975	1025
18	673	706	729	765	837	917	964	997	1049
19	688	722	745	782	856	938	985	1019	1072
20	703	738	762	799	874	958	1007	1042	1096
21	719	754	778	816	893	979	1028	1064	1119
22	734	770	795	833	912	999	1050	1087	1143
23	749	786	811	851	931	1020	1072	1109	1167
24	765	802	828	868	950	1041	1094	1132	1191

[1] Source of weights at each percentile: WHO Child Growth Standards: Length/height-for-age, weight-for-age, weight-for-length, weight-for-height and body mass index-for-age: Methods and development. Geneva, 2006.

Online Supporting Material

Supplemental Table 4. Estimated energy requirements (kcal/d) for boys and girls combined, ages 0-24 months at 5th to 95th WHO 2006 percentiles for body weight

	Weight percentile[1]								
**Month**	**5th**	**10th**	**15th**	**25th**	**50th**	**75th**	**85th**	**90th**	**95th**
0	301	314	323	337	365	394	411	422	439
1	380	397	408	425	459	496	517	531	553
2	467	486	499	519	558	601	625	642	667
3	482	503	517	539	582	629	655	673	702
4	453	475	490	513	559	609	637	657	688
5	484	507	523	548	596	649	679	700	732
6	510	535	552	577	629	684	715	737	771
7	512	537	554	581	635	692	725	748	783
8	533	560	578	606	661	721	755	779	816
9	554	581	600	629	686	748	783	808	847
10	581	609	628	658	717	781	817	843	883
11	597	625	645	675	736	802	840	866	907
12	614	643	663	695	757	825	864	891	933
13	625	655	676	708	772	842	882	910	953
14	642	672	694	726	792	863	904	933	978
15	658	689	711	744	811	884	926	956	1002
16	673	705	728	762	830	905	948	979	1025
17	689	721	744	779	849	926	970	1001	1049
18	704	737	761	797	868	946	992	1023	1073
19	719	753	777	814	887	967	1013	1046	1096
20	734	769	793	831	905	987	1034	1068	1119
21	749	785	809	848	924	1008	1056	1090	1143
22	764	800	826	865	942	1028	1077	1112	1166
23	779	816	842	881	961	1048	1099	1134	1190
24	794	831	858	898	980	1069	1120	1157	1213

[1] Source of weights at each percentile: WHO Child Growth Standards: Length/height-for-age, weight-for-age, weight-for-length, weight-for-height and body mass index-for-age: Methods and development. Geneva, 2006.

Online Supporting Material

Supplemental Table 5. Estimated energy requirements (kcal/d) for boys, ages 0-12 months at 25th, 50th, and 75th WHO 2006 percentiles for body weight, by feeding type

	**Breastfed**			**Formula-fed**		
	**Weight percentile[1]**					
**Month**	**25th**	**50th**	**75th**	**25th**	**50th**	**75th**
0	312	342	373	404	430	459
1	409	445	484	490	522	557
2	521	563	608	591	629	669
3	536	581	630	598	639	682
4	505	554	605	562	605	651
5	544	595	649	596	641	689
6	577	630	687	625	672	722
7	587	642	701	631	680	732
8	612	670	731	653	704	759
9	637	696	759	675	728	784
10	672	733	798	708	762	820
11	691	753	820	724	779	839
12	711	775	844	742	799	860

[1] Source of weights at each percentile: WHO Child Growth Standards: Length/height-for-age, weight-for-age, weight-for-length, weight-for-height and body mass index-for-age: Methods and development. Geneva, 2006.

Online Supporting Material

Supplemental Table 6. Estimated energy requirements (kcal/d) for girls, ages 0-12 months at 25th, 50th, and 75th WHO 2006 percentiles for body weight, by feeding type

	Breastfed			Formula-fed		
	Weight percentile[1]					
**Month**	**25th**	**50th**	**75th**	**25th**	**50th**	**75th**
0	281	309	338	374	399	425
1	368	403	440	452	483	516
2	450	491	534	525	561	600
3	481	526	574	549	589	632
4	465	513	565	527	570	616
5	498	549	605	556	601	651
6	527	581	640	581	629	681
7	528	584	645	579	629	683
8	553	612	676	601	653	710
9	577	637	704	622	676	735
10	602	664	733	645	700	761
11	620	684	755	661	718	781
12	640	706	779	679	737	802

[1] Source of weights at each percentile: WHO Child Growth Standards: Length/height-for-age, weight-for-age, weight-for-length, weight-for-height and body mass index-for-age: Methods and development. Geneva, 2006.

Online Supporting Material

Supplemental Table 7. Estimated energy requirements (kcal/d) for boys and girls combined, ages 0-12 months at 25th, 50th, and 75th WHO 2006 percentiles for body weight, by feeding type

	Breastfed			Formula-fed		
	Weight percentile[1]					
**Month**	**25th**	**50th**	**75th**	**25th**	**50th**	**75th**
0	296	325	356	389	415	442
1	388	424	462	471	502	536
2	486	527	571	558	595	634
3	509	554	602	574	614	657
4	485	533	585	544	587	633
5	521	572	627	576	621	670
6	552	606	663	603	651	702
7	557	613	673	605	654	708
8	583	641	703	627	679	734
9	607	667	731	649	702	760
10	637	698	765	676	731	790
11	655	719	787	692	749	810
12	676	741	811	710	768	831

[1] Source of weights at each percentile: WHO Child Growth Standards: Length/height-for-age, weight-for-age, weight-for-length, weight-for-height and body mass index-for-age: Methods and development. Geneva, 2006.

Online Supporting Material

Supplemental Figure 1. Summary of Systematic Literature Review for studies of Doubly - Labeled Water Technique to measure Total Energy Expenditure in infants, 2000-2019

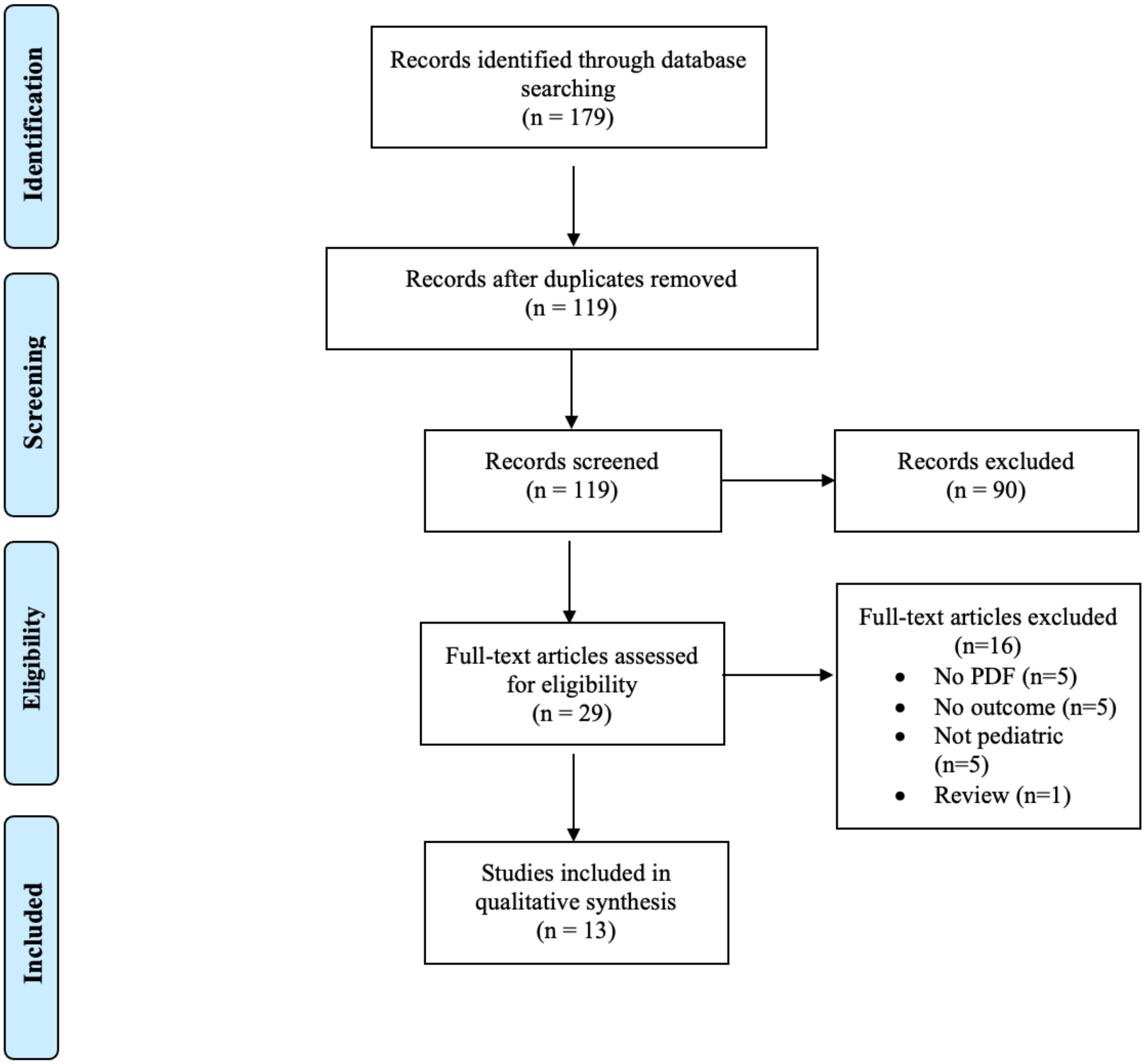

Note: Deduplication in the second step involved the comparison and removal of duplicates from the combined list of hits identified in each individual database search (PubMed, EMBASE, Web of Science, Cochrane Trials, Cochrane Reviews, Clinicaltrials.gov)

Supplemental Figure 2.

Estimated energy requirements at 25th, 50th, and 75th WHO 2006 weight percentiles for age intervals for boys up to 24 months of age

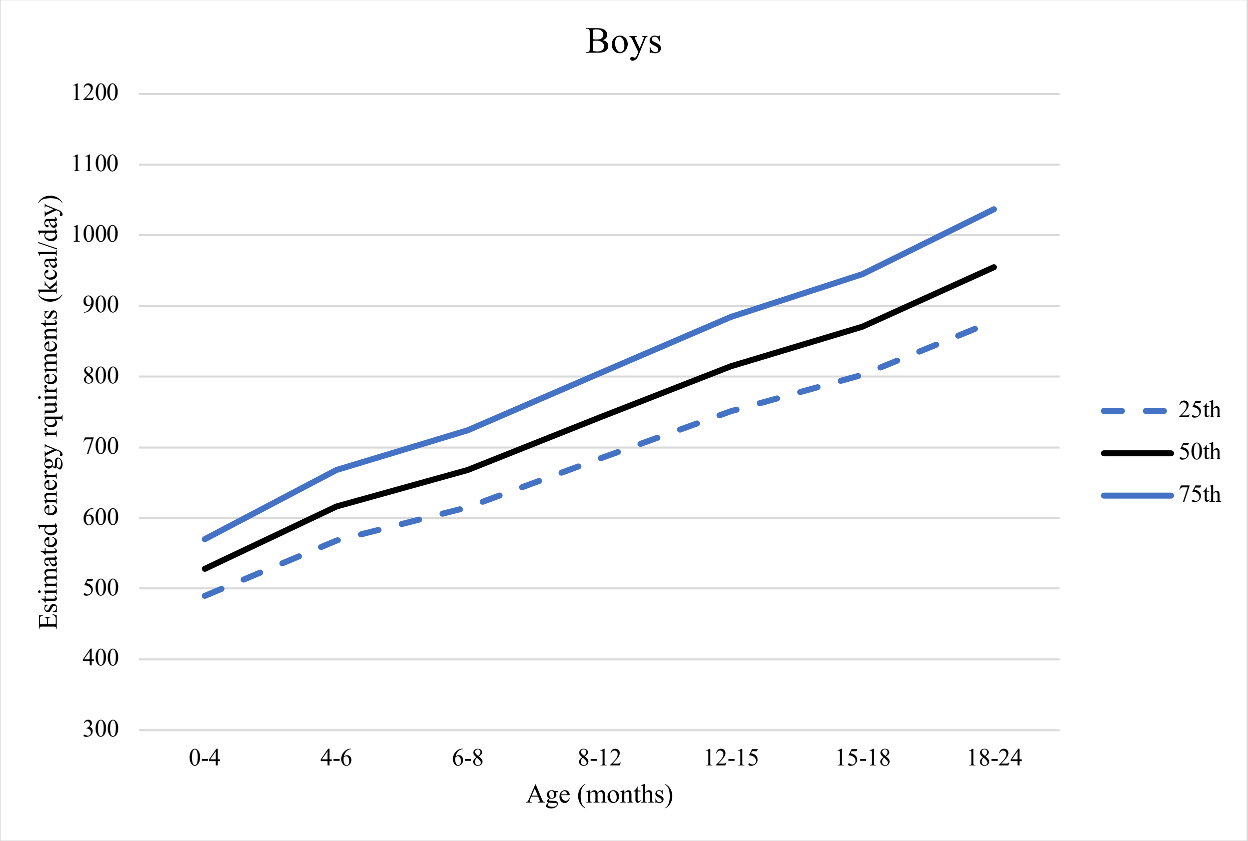

Supplemental Figure 3. Estimated energy requirements at 25th, 50th, and 75th WHO 2006 weight percentiles for age intervals for girls up to 24 months of age

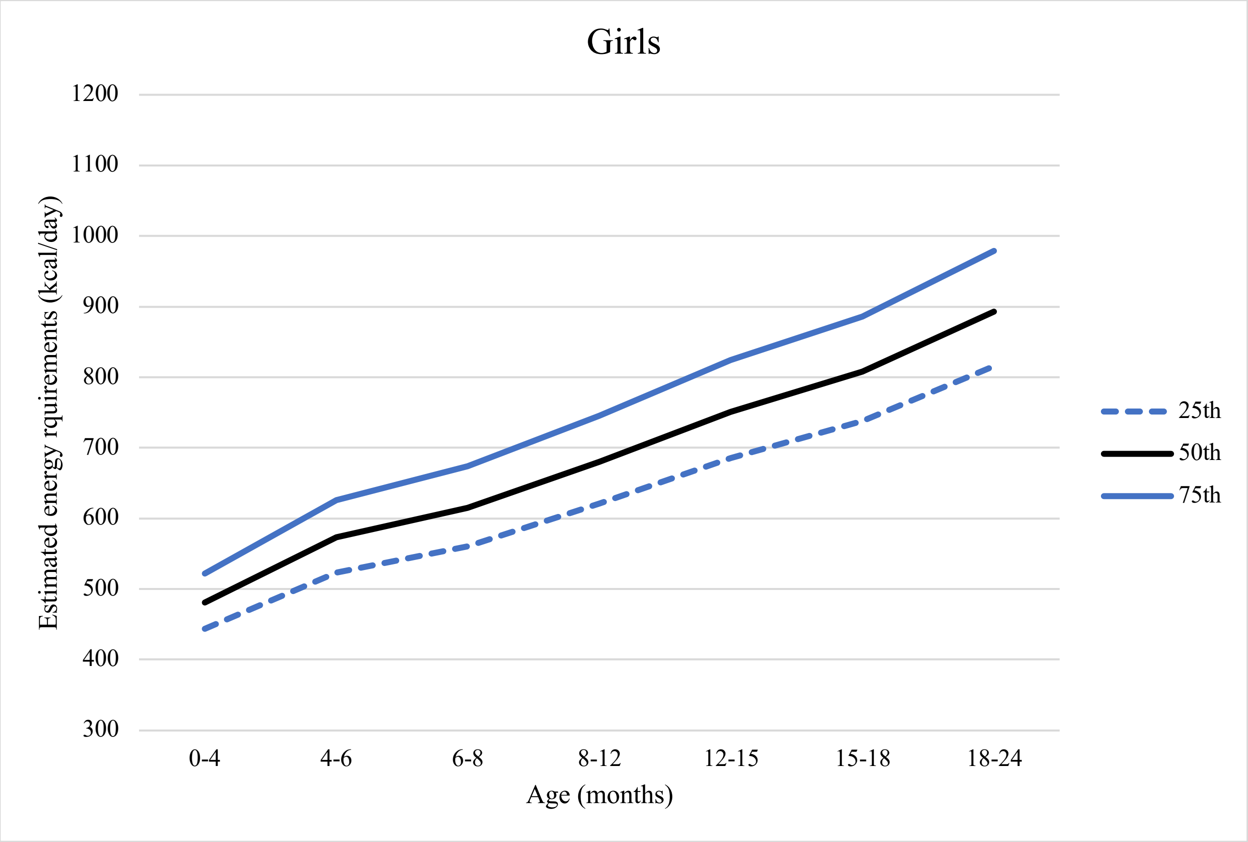

Supplemental Figure 4. Estimated energy requirements over weight percentiles at 25th, 50th, and 75th WHO 2006 weight percentiles for age intervals for boy and girls combined up to 24 months of age

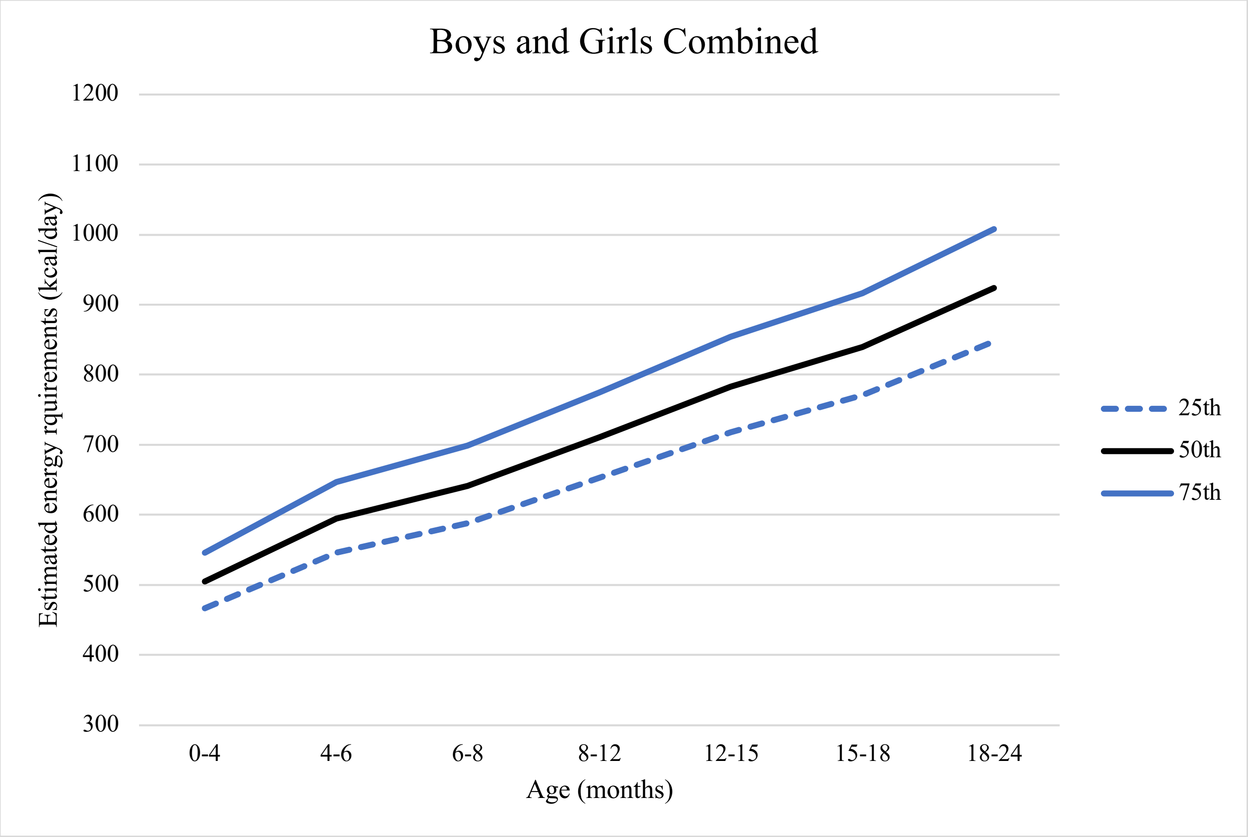

Supplemental Figure 5. IOM model versus meta-regression model estimates for TEE (kcalday) for boys at 25th, 50th, and 75th weight WHO 2006 percentiles

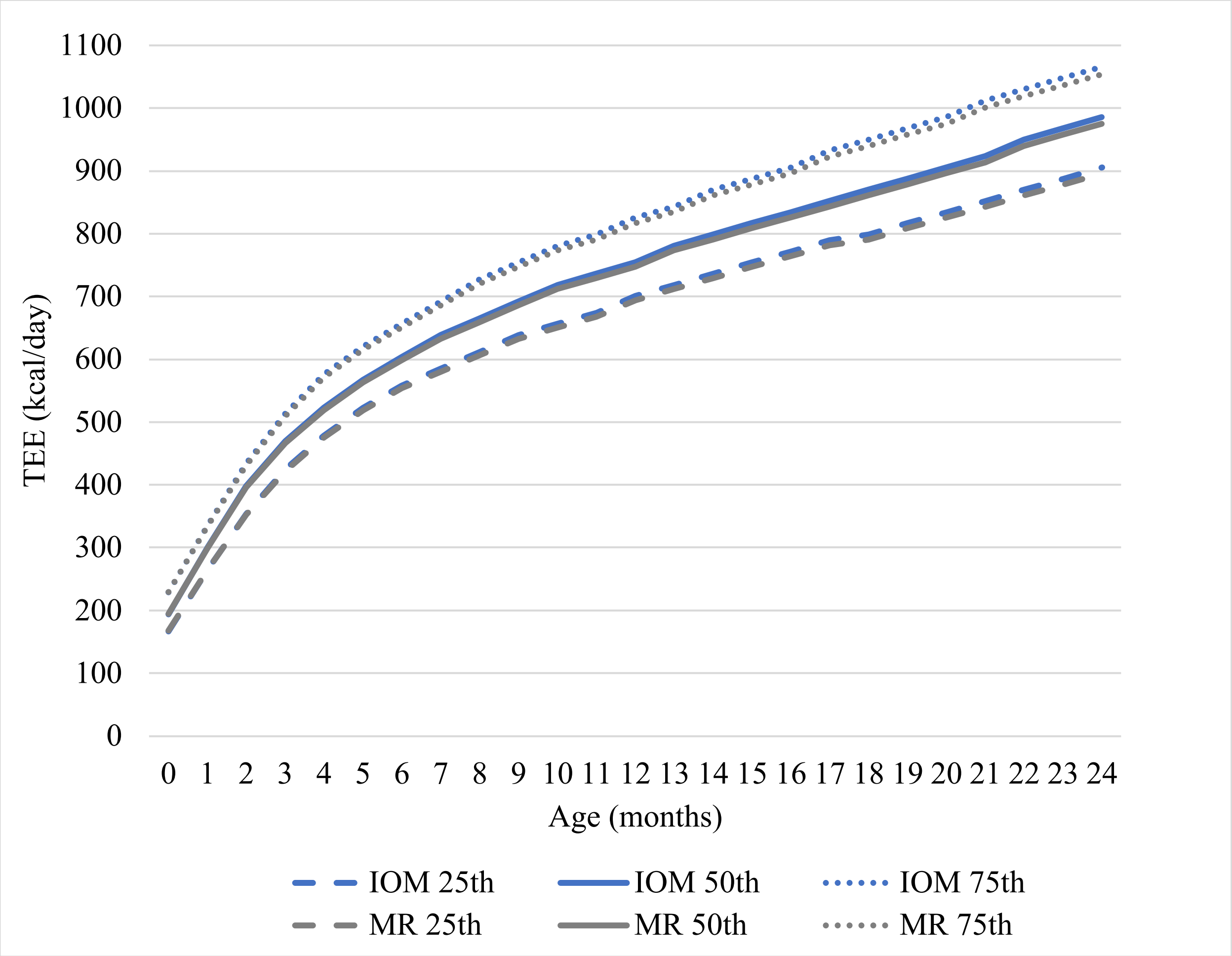

Note: Including the standard errors, the IOM model form is TEE [kcal/day] = 89 (±3) * weight [kg] – 100 (±56). The meta-regression (MR) model form including standard errors is TEE [kcal/day] = 87.7 (±6.1) *weight [kg] – 94.8 (±38.9). Thus, the predicted TEE for a boy at age 12 months at the 50th weight percentile (9.6 kg) is 754 kcal/day based on the IOM model and 747 kcal/day for the meta-regression (95% prediction interval 700-794 kcal/d).

Supplemental Figure 6. IOM model versus meta-regression model estimates for TEE (kcalday) for girls at 25th, 50th, and 75th WHO 2006 weight percentiles

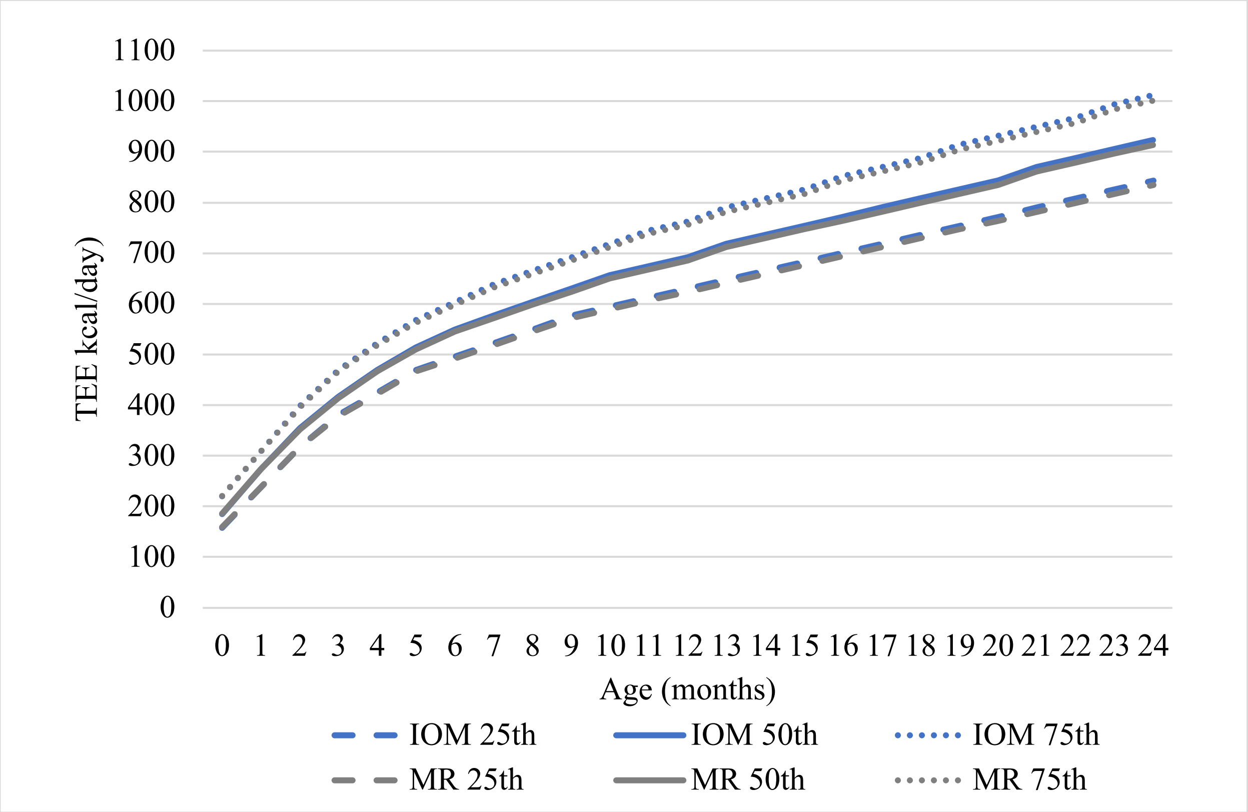

Note: Including the standard errors, the IOM model form is TEE [kcal/day] = 89 (±3) * weight [kg] – 100 (±56). The meta-regression (MR) model form including standard errors is TEE [kcal/day] = 87.7 (±6.1) *weight [kg] – 94.8 (±38.9). Thus, the predicted TEE for a girl at age 12 months at the 50th weight percentile (8.9 kg) is 692 kcal/day based on the IOM model and 686 kcal/day for the meta-regression (95% prediction interval 646-726 kcal/d).
